# Supplementary material for: MAIT cells are functionally impaired in a Mauritian cynomolgus macaque model of SIV and Mtb co-infection
Source: PLoS Pathog. 2020 May 20;16(5):e1008585. doi: 10.1371/journal.ppat.1008585 (PMC7266356; doi:10.1371/journal.ppat.1008585)
Supplement: Attachment — Submitted filename: Ellis-Connell et al cover letter 04 24 20.docx [file ppat.1008585.s004.docx]

April 24th, 2020

Dear Editor,

Here we resubmit our modified manuscript, titled “MAIT cells are functionally impaired in a Mauritian cynomolgus macaque model of SIV and Mtb co-infection.”

We thank the reviewers and the editorial board very much for their careful consideration of our manuscript. We found all of the comments very valuable. We have addressed all of the reviewers’ concerns, which is detailed below in a point-by-point manner. Our responses are highlighted in yellow in the document below. We think the suggested modifications strengthen the manuscript and clarify the interpretation of the data for a more general audience.

Sincerely,

Shelby L. O'Connor, Ph.D.

Associate Professor, UW-Madison

Department of Pathology and Laboratory Medicine

Wisconsin Primate Research Center

Responses to Reviewers’ Questions

**Part I - Summary**
Please use this section to discuss strengths/weaknesses of study, novelty/significance, general execution and scholarship.


Reviewer #1: This manuscript entitled " MAIT cells are functionally impaired in a Mauritian cynomolgus macaque model of SIV and Mtb co-infection " characterizes circulating and lung MAIT cell phenotype and functions in SIV infected and mycobacterium tuberculosis infected macaques in an attempt to understand the impact of SIV infection on MAIT cell functions that could be protective against TB disease. The authors have rigorously conducted the detailed analyses of MAIT cell phenotype and functions in naïve and SIV infected macaques in order to address the impact during co-infection. The authors report that Mtb co-infection in SIV infected macaques contributed to a more activated or exhausted T cell phenotype with elevated levels of PD-1 and TIGIT expression than infection with SIV or Mtb alone. Further, following Mtb infection the MAIT cells from SIV-infected macaques are shown to be functionally impaired in their ability to produce TNFα when compared to SIV-naïve macaques. Overall, the manuscript is very well written and there is a good discussion of the main results, their implications in TB, and most importantly, the caveats of this study.

Reviewer #2: The investigators report the longitudinal effects of SIV (Wisconsin facility), Mtb mono-infection (Pittsburgh facility) and SIV/Mtb co-infection (Pittsburg facility) on macaque MAIT cell abundance and function. Overall, they report low numbers of MAIT cells in peripheral blood, BAL, lymph nodes in all experimental groups utilizing MR1-5OPRU tetramers and analyzing data using both % of T cells and absolute numbers. They also demonstrate that nearly all macaque MAIT cells are CD8+ and can be further subdivided into effector and central memory subsets based on canonical T cell surface receptors CD95 and CD28. They subsequently analyzed these subsets longitudinally ex vivo in peripheral blood (all infection models) and in BAL (SIV mono-infection) and found that MAIT cells transiently upregulate markers of activation/proliferation in the first 4-6 weeks after infection to some degree in all infection models. At the time of necropsy, MAIT cell numbers were found to be increased in the mediastinal lymph node of SIV mono-infected and SIV/Mtb co-infected macaques relative to granulomas. Lymph node MAIT cell number inversely correlated with Mtb CFU. Further, the authors show that mediastinal lymph node MAIT cells express increased activation/exhaustion markers PD1 and TGIT and decreased TNFα, a cytokine associated with control of Mtb infection.

Through a series of in vitro experiments, they demonstrate that healthy or infected macaque peripheral blood MAIT cells respond more robustly to E. coli lysates than those of M. smegmatis. Finally, they demonstrate that MAIT cell TNFα production is impaired after either E. coli or M. smegmatis re-stimulation in vitro in SIV/Mtb co-infected macaques.

Strengths:
This is the first study to compare MAIT cell abundance and function during SIV/Mtb co-infection and mono-infection in macaques. The authors identify distinct MAIT cell subsets based on effector/central memory markers and compare markers of activation/proliferation, chemokine receptors, and transcription factors with non-MAIT CD8+ T cells. They also demonstrate both ex vivo and in vitro functional differences between effector and central memory MAIT cells whose activity is transient during the first weeks after infection to some degree in all experimental models and wanes with no significant proliferation during the adaptive immune response. The authors demonstrate differential MAIT cell activity in vitro to re-stimulation with ligands from E. coli and M. smegmatis and report impaired MAIT cell function in SIV/Mtb co-infected animals.


This manuscript would be of great interest to the general MAIT cell and HIV/TB research community as it rigorously documents longitudinal changes to MAIT cell abundance and function during mono- and co-infection models of SIV and Mtb. The main findings of relatively low numbers of MAIT cells in all groups with transient expression of activation/proliferation markers early after infectious challenge is consistent with previous findings in Mtb mono-infected macaques (Bucson et al. Tuberculosis 2019) demonstrating early, but transient activation/proliferation. This data adds further evidence to support a limited role for MAIT cells in durable immunity against Mtb.

Reviewer #3: This is a well executed study of how MAIT cells in Mauritian cyno macaques are affected by SIV and Mtb infection. With one or two minor exceptions, the data are solid and well presented, and are valuable in their own right.

I have are about MAIT cells, I'm not sure any of the experiments really address the hypothesis (and in fact, doing so in NHPs, with no knockouts and little ability to selectively deplete MAITS, is very difficult). I don't think this undermines the value of the data, which is still strong, but the hypothesis remains one significant quibble about an issue associated with the major hypothesis that drives the paper, include in many places but best stated on lines 268-270 at the beginning of the introduction: "Here, we wanted to test the hypothesis that SIV-dependent dysregulation of MAIT cells weakened their ability to respond to a mycobacterial infection, thus failing to contain Mtb and leading to a rapid early increase in the number of granulomas in co-infected animals." While I think this is a reasonable hypothesis, and despite that all the experiments untested.

We agree with the reviewer that the hypothesis that MAIT cells affect the containment of Mtb infection too difficult to rigorously test without the tools to specifically deplete MAIT cells.

To address this, we have focused our hypothesis instead specifically on the MAIT cells—we hypothesized that SIV infection could affect MAIT cell frequency and/or function within Mtb affected tissues. We have removed any statements indicating that there was a direct effect on the containment of Mtb infection, because we did not test this.

We addressed this in the text, as follows:

The abstract now reads, line 11: “To test the hypothesis that SIV co-infection impairs MAIT cell frequency and function within granulomas, SIV+ and -naïve MCM were infected with….”

Additionally, the abstract reads, line 17: “Our study provides evidence that SIV infection does not prohibit the recruitment of MAIT cells to sites of Mtb infection, but does functionally impair those MAIT cells. Their impaired function could have impacts, either direct or indirect, on the long-term containment of TB disease.”

The author summary reads, line 30: “Overall, our study provides evidence that SIV infection alters MAIT cells phenotypically, and impairs MAIT cell function. What effect this might have on antimycobacterial immunity is an avenue for future exploration.

The introduction reads, line 83: “In this study, we hypothesized that SIV infection would impair MAIT cell frequency and function in Mtb-affected granulomas and LNs….”

Finally, the discussion reads, line 297-298: “Here, we wanted to test the hypothesis that SIV-dependent dysregulation of MAIT cells could impair their frequency and function within Mtb-affected tissues.”

Overall we feel that these changes address the reviewers concerns and make it a much more valid hypothesis that was tested based on the experimental data presented.

**Part II – Major Issues: Key Experiments Required for Acceptance**
Please use this section to detail the key new experiments or modifications of existing experiments that should be absolutely required to validate study conclusions.

Generally, there should be no more than 3 such required experiments or major modifications for a "Major Revision" recommendation. If more than 3 experiments are necessary to validate the study conclusions, then you are encouraged to recommend "Reject".


Reviewer #1: (No Response)

Reviewer #2: Inherent in the macaque model is the small sample size, which inevitably highlights questions of interindividual variability and reproducibility. The SIV mono-infection model and Mtb mono-infection/SIV/Mtb co-infection models were conducted at different facilities with different breeding practices/environmental microbiota which are variables that remain uncontrolled. The authors demonstrate that nearly all macaque MAIT cells are CD8+, but in light of literature identifying both CD4+ and CD4-CD8- MAIT cell subsets in human primates, the manuscript would be strengthened if clearly stated that non-CD8+ MAIT cells were not identified in any macaques. This should also be discussed in the setting of murine animal models where most MAIT cells lack CD4 or CD8 co-expression.

We have now done so in the modified manuscript, beginning with line 101 in the results: All of the circulating MAIT cells were CD8+ in all animals examined, and no CD4+, CD4-CD8-, or CD8+CD4+ MAIT cells were observed (Fig 1A). This was surprising given the fact that in humans and mice, MAIT cells are more diverse…..”

The authors nicely compare immune phenotypes of central/effector memory MAITs with non-MAIT CD8+ T in the first figure. However, this comparison approach was abandoned in later figures where only MAIT cell functional data is reported (eg, fewer TNFα+MAIT detected in SIV/Mtb co-infected mediastinal lymph node MAIT cells). It would greatly strengthen the manuscript if the same functional analyses were performed in non-MAIT CD8+ T cells in order to assess whether this immune dysfunction is unique to the MAIT cell subset or potentially common to conventional CD8+ T cells.

We agree with the reviewer that it would be ideal to compare the function of other non-MAIT CD8+ T cells to the function of the MAIT cells. Our collaborators at the university of Pittburgh are currently working on a manuscript to fully characterize the phenotype and function of conventional CD4 and CD8 T cells in our animal model. Their study will likely include functional analyses of the SIV- and Mtb-specific responses of non-MAIT CD8 T cells before and after Mtb and/or SIV infection. However, we have all agreed that in this MAIT manuscript we can include the functional response to fixed *E.coli* and *M.smegmatis* from the non-MAIT CD8+ T cells. This data was collected in the same set of experiments as the functional assays performed focusing on the MAIT cells in figure 8. Although fixed *E.coli* and *M.smegmatis* are not an ideal stimulus for conventional CD8 T cells, we have found that a range of approximately 2-4% of CD8 T cells do respond to both fixed *E.coli* and *M.smegmatis*. Our results show that at 6 weeks post-Mtb infection for the SIV+ macaques but not the SIV-naïve macaques, there is a longitudinal impairment of TNFα and IFN γ production by the non-MAIT CD8+ T cells in response to both bacterial stimuli.

This suggests that the functional TNFα impairment may be more global, and we agree that it strengthens our manuscript. As a result, we have now decided to include the data describing the functional impairment of CD8 T cells as supplementary figure 3. Text describing this figure can be found on lines 282-292 of the modified manuscript.

Finally, the authors demonstrate that SIV and SIV/Mtb infected macaques have increased numbers of MAIT in mediastinal lymph nodes that inversely correlate with Mtb CFU and that demonstrate upregulated markers of exhaustion and decreased TNFα production. The manuscript would be greatly strengthened if chemokine receptor expression (eg, Figure 1) was characterized on these cells, as this could suggest a mechanism by which SIV may inhibit trafficking to pulmonary sites of infection leading to increased mortality.

We agree once again with the reviewer that this is a very interesting avenue to explore---the idea that SIV infection may impair trafficking of MAIT cells to the sites of Mtb infection in infected lymph nodes is very interesting and worth exploring. Alternatively, it would be interesting to see if Mtb and/or SIV infection could non-specifically activate the MAIT cells and increase the expression of gut homing markers such as α4β7 that would drive MAIT cells away from the lungs and into the gut. We apologize that unfortunately this experiment cannot be performed at this time due to lack of available samples. Instead, we have addressed this in the discussion as a hypothesis that could be tested in the future, beginning line 378 in the modified manuscript.

Reviewer #3: No experiments are required.

**Part III – Minor Issues: Editorial and Data Presentation Modifications**
Please use this section for editorial suggestions as well as relatively minor modifications of existing data that would enhance clarity.


Reviewer #1: Specific comments:

1. The concluding lines in the abstract state that, “the impact of SIV-dependent dysregulation of MAIT cell function on TB disease course was unclear because these animals were euthanized just 6 weeks after Mtb infection. However, their dysregulation by SIV could disrupt control of Mtb replication”. It would be better to focus it on the power of this study, which is that tissue/granuloma specific responses were studied early on during co-infection, rather than stating what was not accomplished due to the study design…It may be nicer to rephrase it focusing on this aspect, for example:
Thus, SIV-mediated dysregulation of MAIT cell functions occurs early following Mtb-coinfection, and has the potential to disrupt control of Mtb replication in the lungs over the course of SIV/TB co-infection.

We thank the reviewer for their comments—we agree that the focus of the manuscript was the dysregulation of MAIT cells by SIV within the tissues during Mtb infection, and along with the comments of another reviewer we have decided to focus our hypothesis on this since we did test it in our study. Our abstract, author summary, introduction, and conclusion have been re-worded in certain places to reflect this (see comments above to reviewer#3).

We have modified the sentence in the abstract, starting with line 17, to read: “Our study provides evidence that SIV infection does not prohibit the recruitment of MAIT cells to sites of Mtb infection, but does functionally impair those MAIT cells. Their impaired function could have impacts, either direct or indirect, on the long-term containment of TB disease.”

2. Line 73 in Introduction section, the rationale for using Mauritian cynomolgus macaques (MCM) is given as having simplified major histocompatibility complex (MHC) genetics. The authors need to clarify on why that is an advantage for this study to make it easier for readers from outside the nonhuman primate (nhp) field to understand the rationale, especially since most other nhp studies have used Indian rhesus macaques or the Chinese cynomolgus macaques.

We agree that our introduction did not fully describe the animal model we use, and we have modified the introduction to explain in more detail that the simplified MHC genetics means that all animals in the study will have similar CD4 and CD8 T cell responses. The simplified MHC genetics previously allowed us to generate tetramer reagents to characterize the adaptive immune response. We have modified the text in the introduction beginning with line 75-78: “Because of the simplified genetics, the adaptive immune response is much easier to characterize….”

3. There is a lack of consistency in the gating for MAIT cells between figures 1, 3 and 5. Fig.1 B shows distribution of TCR Va7.2 and CD4 and CD8 on MR1 tet+ cells and the tet- T cells. Please label the y-axis as %TCR Va7.2 instead of TCR only. Also, it is not clear that the % CD4 or CD8 expression inside the box on the right is from total CD3+ tet+ cells, or MR1tet+TCRVα7.2+ cells, although the legend mentions the latter. It would be simpler to just add a label as MR1tet+TCRVα7.2+ cells on the box that shows CD4, CD8 expression and memory phenotype. Based on Fig 1, the MAIT cells are from CD3+ MR1 5OPRU tet+ cells (Fig 1 A, B). Then in Fig 3, 4 and 6, the MAIT cells are gated on CD8+ cells instead of CD3+ MR1 5OPRU tet+ cells shown in the top panel of Fig 3. Although all of the tet+ cells are in the CD8hi population, the denominator (total CD3 cells or CD8+ T cells) can impact the cell counts, especially in SIV infection when CD4/CD8 ratios change. In Fig 5, we are back to CD3+ MR1 5OPRU tet+ cells. It would be good to stick to one gating strategy to define MAIT cells throughout the study.

We agree that the gating is very unclear for Figure 1. In Figure 1B-C, the phenotypes of the memory cells were actually based on CD8+MR15OPRUtet+ cells, and we realize that this was not mentioned and is not evident at all from the image in 1A.

To make the gating strategy of Figure 1 clearer, we are now showing the flow plots for what was gated on the CD3+MR1tet+ parent gate (CD4, CD8, and TCRVα7.2 expression) as part of figure 1A. Then, for Figure 1B we now show the phenotypic characterization of the CD8+MR1tet+ cells with regards to their frequency in the peripheral blood of the SIV-naïve MCM, as well as the characterization of memory markers CD28 and CD95.

Figures 1C and 1D remain the same, with the exception of more clearly labeling that the markers shown on the conventional (MR1tet-) and MAIT (MR1tet+) memory cells are based on the CD8+ parent gate.

All of these changes have also been reflected in the results section, modified manuscript lines 101-111.

For Figure 3, we agree that the gating schematic was unclear. We have changed Figure 3A to make it more evident that all of the PBMC analysis for figure 3 was performed on CD8+MR1tet+ cells.

Since all of our phenotyping/functional assays were collected on CD8+MR1tet+ cells for the remainder of the manuscript, it is logical to stick to a CD8+MR1tet+ gate, and we have modified Figure 5 to focus on the MAIT cells within the CD8+ parent gate instead of the MAIT cells within the CD3+ parent gate. We are now showing the frequency of CD8+MR1tet+ cells in Figure 5B, middle panel and also for Figure 5C, instead of CD3+MR1tet+ cells.

We apologize for this lack of clarity with our gating schematics, and hopefully with these changes it will now be more evident that we performed our analyses on the CD8+MR1tet+ cells.

Reviewer #2: (No Response)

Reviewer #3: I'll give these in two bullet lists, one about figures, and one about text.

Notes on Figures:

* Figure 1A - on the right most panel, please plot MAIT frequency vs age

We have added this to the modified figure 1 as part of Figure 1B. There were no correlations between MAIT frequencies and ages of the animals, but this is good to note.

* Figure 1B - on right most plot, replace bar graph with dots and quartiles

We have changed the bar graphs to dots/quartile graphs as part of the modified Figure 1 A and B.

* Figure 3B, C, D - add color coding to symbols

For the sake of reserving color-coding for key figures where it was important to differentiate between groups (such as SIV/Mtb and Mtb only groups) we would prefer to leave Figures 3B, C, and D as black lines.

* Figure 4A highlights LN and granuloma collection, but the data in the rest of the figure appear to be about PBMC. This is just a bit strange organizationally. It looks like all the LN and granuloma stuff will be analyzed in a future manuscript.

We agree with the reviewer that keeping the sample collection outline as it currently stands as a part of Figure 4 might be a bit confusing to a reader. We still would like to leave all of the different samples we collected in the manuscript as we did examine MAIT cells in blood (Figure 4), BAL (Figure 5) and granulomas and LN (Figure 6). Therefore, we have removed the pictures of the granulomas and LN from Figure 4 to keep the focus completely about blood collection. Then, we have also modified Figure 6 to add the images of the granulomas and LN collection. This should hopefully make it very clear what was being analyzed within each figure.

* Figure 5. Add comparisons to blood phenotypes if data are available. Explore a looser gate on FSC-SSC to catch potential blasts.

We have added the comparisons for ki67, CD39, PD1, TIGIT, and HLADR on CD8+MAIT+ cells for blood and BAL for each timepoint as a part of a new supplementary Figure 1. We did find that others have compared the PD-1, ki67, and HLADR phenotypes of conventional CD4 and CD8 T cells in blood and BAL for HIV+ individuals and our data are consistent with those findings for the SIV+ macaques. We have discussed our findings as well as comparisons between the findings of others in the modified manuscript, lines 190-197.

* Figure 7: I’d like to see sample a flow cytometry plots of MR1tet+ vs each of CD107, IFNg, and TNFα gated on CD8+ cells corresponding to data in panel A.

We agree that adding the flow cytometry for the functional assays is helpful, and so we have added an example of some of the staining as a supplementary figure 2 for simplicity. The reference to supplementary figure 2 can be found in the modified manuscript, lines 245-247.

* Figure 7: are there complications when comparing E. Coli vs M.smeg stimulations, including potential differential stimulation of innate cytokine responses that have nothing to do with MR1-restricted recognition. In addition, the PBMC used were frozen — this probably makes a difference with monocyte APC function. There might also be a complication with MR1 expression and MAITs responding to cytokines rather than riboflavin precursors. Also, protocol as written is flawed because monensin is added at the same time as bacterial -- this is likely to limit transit of MR1 to the cell surface.

We realize that there was an oversight in our methods---the bacteria were added to the cells for approximately 90 minutes prior to the addition of brefeldin A and monensin, and this is not explained in our methods. We have fixed this error in the modified manuscript, line 545. We thank the reviewer for their careful attention to detail in noticing this.

We agree that there are many other caveats to the comparison of the *E.coli* and *M.smegmatis* stimuli. It was not our intention to compare the two stimuli, merely to have two different bacteria (one commonly used bacterial stimulation, and one mycobacterial) to analyze MAIT cell function longitudinally.

To address some of these caveats, we have added a paragraph to the discussion, lines 340-352, emphasizing that the bacteria may cause differential stimulation of innate immune cells leading to the production of cytokines that may result in variable degrees of TCR-independent activation of MAIT cells. We also included in the paragraph the very good point that these functional assays were performed on frozen PBMC which we agree likely affects the outcome of the assay and the antigen presenting capabilities of the APC present in the sample. Due to our limited amounts of PBMC we were unable to grow up monocytes to use as APCs for these experiments, but future studies should use autologous monocytes for a more careful analysis.

Notes on Text

* Line 20: The statement here (and similar ones elsewhere) makes an assumption not proven in the manuscript, that MAIT cells contribute to control of Mtb. (Also discussed above)

We have modified the hypothesis of our manuscript to match the results and have addressed these concerns. Please see our above comments as to where in the manuscript we have modified the text.

* Line 51: substitute “invariant” for “innate” (which is a debatable term here).

On line 51, the word is already “innate,” did the reviewer mean the opposite? We agree that the use of the word innate to describe MAIT cells could be not entirely accurate, particularly for this sentence. Therefore, to avoid using the word “innate,” we have changed the sentence in the modified manuscript, line 50, to read “MAIT cells are a specialized population of T lymphocytic cells….”

* The paragraph beginning on Line 61 doesn’t indicate why a population specific for riboflavin precursors should be affected by HIV or SIV infection.

We did not address the mechanism by which this might be occurring, but several studies do indicate that MAIT cells might be either depleted or phenotypically altered by HIV/SIV infection. Furthermore, MAIT cells are functionally impaired in chronic HIV infection and more subject to apoptosis. To address this, we have modified the sentence in the manuscript to read, beginning line 61: “Several cross-sectional human studies indicate that MAIT cells are depleted during HIV infection, but the mechanism by which this occurs is not well understood. The depletion has been suggested to occur from immune over-activation and subsequent apoptosis, as well as possibly via downmodulation of surface receptors.”

* Lines 74-77. The connection between granuloma numbers and impaired immune responses is not obvious.

We agree, and we have modified the text to read beginning on line 80-82: “This observation suggested that SIV infection impaired the ability of the co-infected MCM to contain Mtb infection some time between 4 and 8 weeks post-Mtb challenge.”

* Line 134-137. Were increases in markers such as Ki67 due to cytokine receptors on MAITs or TCR? Probably hard to determine in MCM, but this fact should be noted.

We did not determine if the upregulation of activation markers was occurring in a TCR dependent or independent manner. We have added a sentence to the results section to address this, beginning on line 146-149: “We did not assess if the activation of MAIT cells or their increased expression of ki67 was occurring in a TCRVα7.2-dependent or -independent manner…..”

* Line 145-146. Specify Mtb strain within the text here.*

We have added this, line 156 of the modified manuscript.

Lines 236-236 could use a reference for the pHRODO assay.

We have modified this to address this concern, beginning line 252-255 of the modified manuscript, “We labeled equal amounts of *M. smegmatis* and *E. coli* with a pHRODO^TM^ green dye which increases in fluorescence intensity when labeled bacteria are taken up by antigen presenting cells, and has been used previously……”

* Line 290. The relationship between MAIT derived TNFα production and Mtb control is not established by this paper. Could be other T cells. This whole paragraph should be toned down a bit. This is acknowledged on line 311.

At the request of another reviewer, we have added increased supporting evidence that the defect in TNFα production may be occurring in more than just MAIT cells. As part of the same set of experiments, we also found that conventional CD8 T cells (CD8+MR1tet-) were also impaired in their ability to produce TNFα at six weeks post Mtb infection in the SIV/Mtb co-infected cohort. Similar caveats exist to this experiment as with the MAIT cell experiment (we used frozen PBMC). However, our collaborators at the university of Pittsburgh are also exploring defects in the conventional T cell response in a separate manuscript.

However, overall this data is still only preliminary and does not address any mechanism by which this might be occurring, nor does it prove that the decrease in TNFα production by MAIT cells causes the loss of containment of Mtb. We have toned down the paragraph in the following locations:

Line 318: “Similar defects were observed in TNFα production from conventional CD8 T cells in the same in vitro assays (supplementary Fig 3). Despite this, the decreased production of TNFα by MAIT cells alone is very unlikely to be the only determinant for containment of Mtb infection.”

We have also removed the following sentence from the paragraph: “Our data are thus consistent with the hypothesis that defective TNFα production in SIV+ MCM contributes to the rapid dissemination of granulomas previously observed in SIV+ macaques between 4 and 8 weeks after Mtb infection.”
